# Immunological role and prognostic value of the SKA family in pan-cancer analysis

**DOI:** 10.3389/fimmu.2023.1012999

**Published:** 2023-04-26

**Authors:** Zhengtian Li, Lanying Huang, Jiachen Li, Wenkang Yang, Weichao Li, Qiuzhong Long, Xinyu Dai, Hongtao Wang, Gang Du

**Affiliations:** ^1^ Department of Bone and Joint Surgery, The First Affiliated Hospital of Guangxi Medical University, Nanning, China; ^2^ Department of Rehabilitation Medicine, The First Affiliated Hospital of Guangxi Medical University, Nanning, China; ^3^ Department of Gastrointestinal and Gland Surgery, The First Affiliated Hospital of Guangxi Medical University, Nanning, China

**Keywords:** SKA family, pan-cancer, prognosis, tumor microenvironment, immunotherapy response

## Abstract

**Background:**

The spindle and kinetochore associated (SKA) complex, which plays important roles in proper chromosome segregation during mitosis by maintaining the stabilization of kinetochore-spindle microtubule attachment during mitosis, has recently been reported to exert regulatory effects on the initiation and progression of various human cancer types. Nevertheless, the prognostic significance and immune infiltration of the SKA family across cancers have not been well elucidated.

**Methods:**

Using data from three large public datasets, including The Cancer Genome Atlas, Genotype-Tissue Expression, and Gene Expression Omnibus databases, a novel scoring system (termed the SKA score) was developed to quantify the SKA family level across cancers. We then evaluated the prognostic impact of the SKA score on survival and assessed the effect of the SKA score on immunotherapy at the pan-cancer level using multiomics bioinformatic analyses. The correlation of the SKA score and the tumor microenvironment (TME) was also explored in depth. Potential small molecular compounds and chemotherapeutic agents were assessed by CTRP and GDSC analyses. Immunohistochemistry was performed to verify the expression of the SKA family genes.

**Results:**

Our results demonstrated a close correlation between the SKA score and tumor development and prognosis in multiple cancers. The SKA score was positively related to cell cycle pathways and DNA replication across cancers, such as E2F targets, the G2M checkpoint, MYC targets V1/V2, mitotic spindles and DNA repair. Additionally, the SKA score was negatively related to the infiltration of various immune cells with antitumor effects in the TME. In addition, the potential value of the SKA score was identified to predict immunotherapy response for melanoma and bladder cancer. We also demonstrated a correlation between SKA1/2/3 and the response to drug treatment across cancers and the promising potential of the SKA complex and its genes as therapeutic targets in cancer. Immunohistochemistry demonstrated that the expression differences of SKA1/2/3 were significant between the breast cancer group and the paracancerous group.

**Conclusion:**

The SKA score plays a critical role in 33 cancer types and is highly related to tumor prognosis. Patients with elevated SKA scores have a clear immunosuppressive TME. The SKA score may serve as a predictor for patients receiving anti-PD-1/L1 therapy.

## Introduction

SKA1, along with SKA2 and SKA3, constitutes the spindle- and kinetochore-associated subunit complex. The SKA family is an indispensable element that stabilizes spindle microtubules attaching to the kinetochore (KT) and ensures the completion of the mitosis process (1–4). Notably, dysregulated expression of SKA1/2/3 was involved in the tumorigenesis and progression of multiple cancer types such as liver cancer, breast cancer, cervical cancer and other malignant tumors (5–7), which in turn can affect the prognosis of cancer patients. High SKA1 expression contributed to the development and progression of hepatocellular carcinoma and reflected unfavorable prognosis (3). In breast cancer (BC), SKA2 is highly expressed and serves as a useful biomarker in both tumor initiation and progression (4). In addition, a high level of SKA3 was closely related to the cellular growth, proliferation, invasion and metastasis of cervical cancer cells and thus linked to poor prognosis (5). To date, however, most studies on SKA1/2/3 in cancers are based on a single tumor or single gene. The potential value for the clinical application of SKA1/2/3, especially in the area of prognosis and the functional role of pan-cancer, remains to be explored.

Around or inside the malignant tumor, stromal cells, endothelial cells, intrinsic cells, lymphocytes and their secreted active mediators exist, which can interact with malignant tumor cells, together forming a unique tumor microenvironment (TME) (8). There is a growing body of studies supporting the important role of the TME in tumorigenesis, tumor progression, and treatment efficacy (9, 10). Other studies have shown that immune cell infiltration in the TME is associated with the immunotherapy response (11). For instance, tumor-associated macrophages (TAMs) can serve as a cancer promoter and reflect an adverse prognosis for cancers by secreting molecules stimulating tumor growth (12, 13). In contrast, CD8+ and CD4+ T cells in the TME exert antitumor effects by recognizing and clearing dysplastic cancer cells, which are highly related to survival outcomes and immunotherapeutic responses in cancer patients (14). In addition, immune checkpoint inhibitors (ICIs) have recently been shown to be a promising therapy method in advanced cancer patients (15–17). Inhibition of immune checkpoint targets, including PD-1/PD-L1, is clinically effective in a variety of cancers (18). However, ICI therapy only benefits a small portion of cancer patients due to its low response rates. Therefore, the identification of reliable biomarkers for screening immunotherapy candidates and therapeutic targets in cancers is challenging and promising.

In this study, we conducted a comprehensive analysis of SKA1/2/3 at the pan-cancer level, including gene alterations (mutation, copy number, methylation), expression, prognostic value, pathway enrichment, effects on the tumor microenvironment (TME) and immunotherapy. We further established a scoring system (termed the SKA score) across cancers to provide more accurate prognostic information for individual patients. Our study specifically focuses on the possible impact of the SKA score in cancer and sheds new light on the biological roles of the SKA complex and the potential of the SKA score as a therapeutic response indicator and target for anticancer treatment.

## Materials and methods

### Data collection

Public data, including the RNA-seq matrix and corresponding clinical information of The Cancer Genome Atlas (TCGA) and Genotype-Tissue Expression (GTEx), were downloaded from the UCSC Xena website (https://xenabrowser.net/datapages/) (19). Immune infiltration data were collected from the Immune Cell Abundance Identifier (ImmuCellAI) (20) (http://bioinfo.life.hust.edu.cn/ImmuCellAI#!/) and TIMER2 databases (21) (http://timer.cistrome.org/) and matched with the clinical information downloaded from the UCSC Xena. The immunotherapy-related independent datasets GSE91061 and IMvigor210 were obtained from the Gene Expression Omnibus (GEO) database (https://www.ncbi.nlm.nih.gov) and the website based on the Creative Commons 3.0 license (http://research-pub.Gene.com/imvigor210corebiologies).

### Differentially expressed gene analysis

First, we investigated the expression heterogeneity of SKA family-related genes between tumor and nontumor tissues in 31 types of cancers by the “limma” package (22). The obtained results are displayed in a heatmap, with P >= 0.05 set as white, red for high expression, blue for low expression, and numerical values representing logFC values. A functional protein association network was constructed for SKA1/2/3 using the STRING tool with high confidence (23).

### Somatic copy number alteration and mutation analysis

To investigate the correlation between the mRNA expression and genomic alterations of the SKA family, we performed Spearman’s rank correlation analysis based on the Gene Set Cancer Analysis (GSCA) database (24) (http://bioinfo.life.hust.edu.cn/GSCA/#/). GSCA is an integrated database analysis platform that supports the visualization of genomic and immunogenomic gene set cancer analysis. Data including single nucleotide variation (SNV), amplification, homozygous and heterozygous deletion and amplification were adopted for the assessment and analysis of the gene mutation status of SKA family genes. Spearman’s correlation analysis was performed to determine the strength of associations.

### DNA methylation analysis

By mining the UCSC database, we obtained DNA methylation (Illumina Human Methylation 450) data from 33 different types of tumors for further analyses. For some tumors, Infinium Human Methylation 450 data were lacking. Differential methylation of 13 pairs of cancerous tissues and normal tissues was analyzed by the Wilcoxon rank test. Genes with a P-value < 0.05 and logFC < 0 were regarded as hypomethylated, and those with a P-value < 0.05 and logFC > 0 were regarded as hypermethylated. Spearman’s correlation analysis was performed to determine the strength of the association between methylation and gene expression levels. P < 0.05 was considered significant. All results were generated from the GSCA online database and presented as dot plots.

### Establishment of the SKA score

To assess the prognostic value of the SKA family, the R (version 4.1.1) “GSVA” package was used to perform single-sample gene set enrichment analysis (ssGSEA) (25) to calculate the SKA score of each sample across 33 cancer types. The following is the basic principle of the specific SKA score generation: ssGSEA is a function in the R package “GSVA”. For the pan-cancer gene expression matrix, ssGSEA first sorted the expression levels of all genes in the sample to obtain its rank among all genes. Then, for the input SKA family gene set (includes SKA1, SKA2 and SKA3), the genes present in the expression data are found from the gene set and counted, and the expression levels of these genes are summed. Then, based on the abovementioned evaluation, the enrichment score of each gene in the pathway was calculated, and the gene sequence was further disrupted to recalculate the enrichment score, which was repeated 1000 times. Finally, the enrichment score was generated according to the empirical cumulative distribution function (ECDF), that is, the SKA score. Each sample in pan-cancer has an SKA score value. The corresponding R code is provided in the **Supplementary Material** (**Table S1**).

### Prognostic analysis of SKA score

To analyze the effect of the SKA score on the survival outcomes of patients, such as overall survival (OS), disease-specific survival (DSS), disease-free interval (DFI), and progression-free interval (PFI), we performed univariate Cox regression (UniCox) analyses across 33 cancer types based on the R “survminer” and “survival” packages. We performed Cox proportional hazards regression analyses and determined the hazard ratio (HR) with 95% confidence intervals and corresponding P-values to estimate the prognostic value of the SKA score in each cancer. The results were represented by a heatmap using the R “ggplot2” package. The Kaplan–Meier (KM) method was used to investigate the prognostic value of the SKA score in human cancers using the R packages limma, survival, and survminer. P < 0.05 was considered significant.

### Gene enrichment analysis

To investigate the biological functions of SKA1/2/3 and its role in 33 cancer types, the R “GSVA” package (26) was used to perform gene set variation analysis (GSVA) enrichment analysis to assess the association between the SKA score and 50 HALLMARK pathways in 33 cancer types according to the MSigDB database (27) (http://software.broadinstitute.org/gsea/msigdb/index.jsp).

### Tumor microenvironment analysis

The TME score, including stromal score, immune score, ESTIMATE score, and tumor purity score, was computed for each patient in 33 cancer types using the R “ESTIMATE” package. In subsequent analysis, we further explored the correlation between the SKA score and these scores. For further validation, we obtained TME-related pathways from a previous study (28), which have been widely used in omics data analysis, and then calculated corresponding pathway scores to explore the correlation of the SKA score with immune cell infiltration at the pan-cancer level. The obtained results were displayed using a heatmap with the help of the R “ggplot2” package.

### Drug sensitivity analysis

To assess the correlations between the SKA family genes and small-molecule drugs, we computed the Pearson correlation coefficients between SKA1/2/3 and the drug sensitivity percentage using the Genomics of Drug Sensitivity in Cancer (GDSC-https://www.sanger.ac.uk/tool/gdsc-genomics-drug-sensitivity-cancer/) (29) and Cancer Therapeutics Response Portal (CTRP-https://portals.broadinstitute.org/ctrp/) datasets.

### Immunohistochemistry

We obtained five breast cancer (BC) or five gastric cancer (GC) and five paracancerous samples from The First Affiliated Hospital of Guangxi Medical University for immunohistochemistry. All patients were diagnosed as BC or GC by pathology. Clinicopathological data, such as sex and age, were collected. The study was conducted in accordance with the Declaration of Helsinki. This study was approved by ethics committee of the First Affiliated Hospital of Guangxi Medical University, Nanning, People’s Republic of China. Anti-SKA1/2/3 antibody were purchased from Abcam website (Cambridge, MA). Immunohistochemical staining method followed the instructions of manufacturers. Ten visual fields(Scale bar=200μm) were randomly selected, and two researchers independently read the images. Hematoxylin stained the cell nucleus blue, and positive expression of diaminobenzidine (DAB) is brownish yellow.

### Statistical analysis

All data are expressed as the mean ± standard deviation (SD) unless indicated otherwise. Student’s t-test or analysis of variance (ANOVA) was used to determine the differences among groups. For patients with the same ID, those ending in -01 are tumor tissues, and those ending in -11 are paired normal tissues. The method used is the paired t-test. The R software package (version 4.1.1, https://www.r-project.org/) was used for the statistical analysis. All correlations were performed with Pearson’s correlation r, unless otherwise stated, and the results are mainly displayed in the heatmaps. The differences were considered statistically significant when P < 0.05 and were reported as follows: ****P < 0.0001, ***P < 0.001, **P < 0.01, *P < 0.05.

## Results

### mRNA level and prognostic value of the SKA family

**Figure 1:**
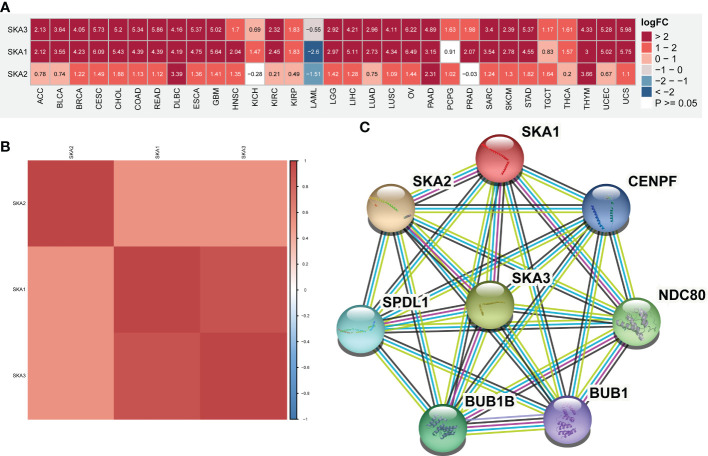
mRNA expression of SKA1/2/3 across cancers. **(A)** Heatmap depicting the differential expression information (including Log fold-change and P value) of SKA1/2/3 at the pan-cancer level. **(B)** The Spearman correlation between SKA1, SKA2, and SKA3 is shown in the heatmap. The darker is the red color, the stronger is the correlation between genes. **(C)** The protein–protein interaction (PPI) networks among SKA1/2/3 proteins.

**Figure 2:**
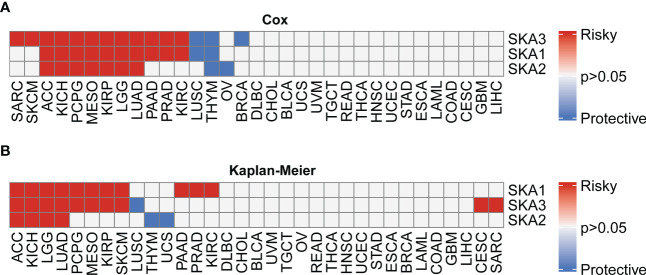
Univariate Cox (uniCox) analysis and prognostic value of the mRNA expression of distinct SKA family members across cancers. **(A)** Heatmap depicting the uniCox results of SKA family members across cancers. **(B)** Kaplan–Meier curve analyses showed that the overexpression of SKA family genes in multiple cancer types was associated with poor prognosis. For univariate Cox regression analysis results, P > 0.05 are all gray; p < 0.05, HR > 1 is defined as “Risky”, and p < 0.05, HR < 1 is defined as “Protective”. For Kaplan–Meir analysis results, P > 0.05 are all gray; p<0.05, poor prognosis is defined as “Risky”, good prognosis is defined as “Protective”.

First, based on the TCGA and GTEx databases, for each SKA family gene, we explored the differential expression across 33 cancer types. **Table S2** lists the 33 cancer types, their abbreviations and sample size for each cancer type. Because normal tissue for UVM and MESO was absent in TCGA and GTEx, these two cancer types for which differential analysis was not performed are not shown in the heatmap. The expression of 3 SKA family genes differed among 31 cancer types, as shown in **Figure 1A**. From this figure, we know that the SKA genes were highly expressed in the 30 cancer types, except for LAML. We speculate that SKA gene expression in LAML in this cancer type is quite different from that in other tumor types, which may be due to its hematological disease. Further analysis of TCGA pan-cancer data revealed a correlation between SKA family genes (**Figure 1B**). In **Figure 1C**, we preliminarily predicted the protein interactions of SKA1/2/3 through the String website. After reviewing the related literatures, we had learned some of the functions of these genes related to SKA family genes in cancer. CENPF could promote the progression of papillary thyroid carcinoma by affecting cell proliferation and apoptosis and the high expression (30) of CENPF was associated with the poor prognosis of breast cancer and bone metastasis (31). Kodama et al. found that SPDL1 can be regulated by MRTFB to inhibit the development of colorectal cancer (32). Zeng et al. found that the expression level of NDC80 in human glioblastoma cells was significantly higher than that in normal cells and that NDC80 could promote the proliferation and invasion of human glioblastoma cells (33). Qin et al. also found that BUB1B is highly expressed in nasopharyngeal carcinoma and that BUB1B can promote tumor progression by regulating the cell cycle (34). Zhu et al. found that BUB1 promoted the proliferation of liver cancer cells by activating the phosphorylation of SMAD2 (35). The above results shown that these genes also played potential roles in some types of cancer and the specific pathways or functions involved in these genes deserved further exploration in the future. In addition, we conducted a univariate analysis of each SKA family gene in 33 tumors (**Figure 2A**). Three genes in the SKA family were shown to be risk factors in tumors, including ACC, KICH, PCPG, MESO, KIRP, LGG, and LUAD. Kaplan–Meier curve analyses showed that the overexpression of SKA family genes in multiple cancer types, such as ACC, KICH, LGG, and LUAD, was related to a poor prognosis (**Figure 2B**).

### Gene alterations of the SKA family

**Figure 3:**
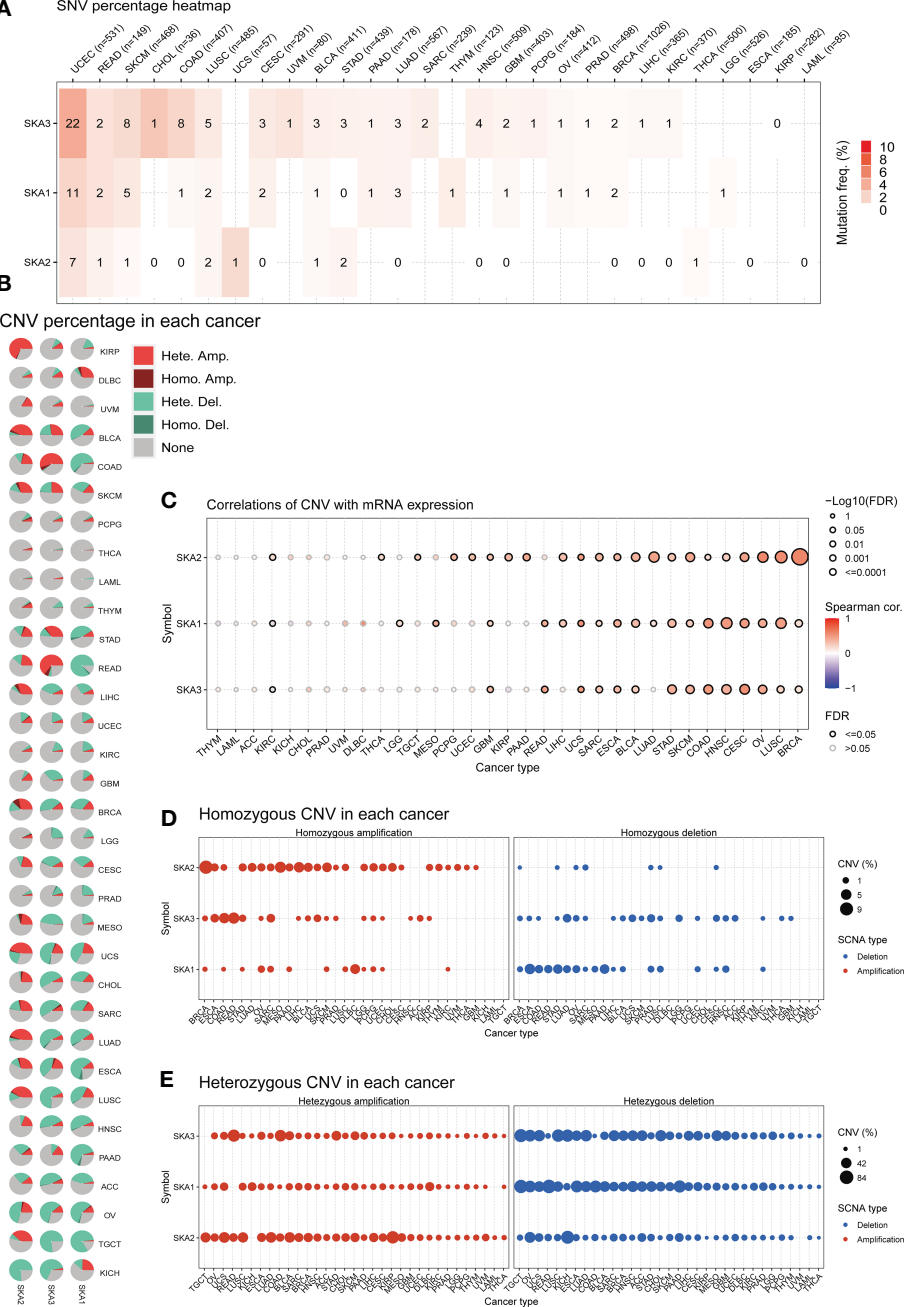
Gene alterations in SKA1/2/3 across 33 cancer types. **(A)** The frequency landscape of deleterious mutations in 33 tumor types. **(B)** The CNV information of SKA1/2/3 in pan-cancer is summarized in the pie chart. **(C)** The relationship of CNV with the mRNA levels of SKA1/2/3. **(D, E)** The percentage of homozygous **(D)** or heterozygous **(E)** CNV (including homozygous amplification, homozygous deletion, heterozygous amplification, and heterozygous deletion) for each SKA member in pan-cancer.

**Figure 4:**
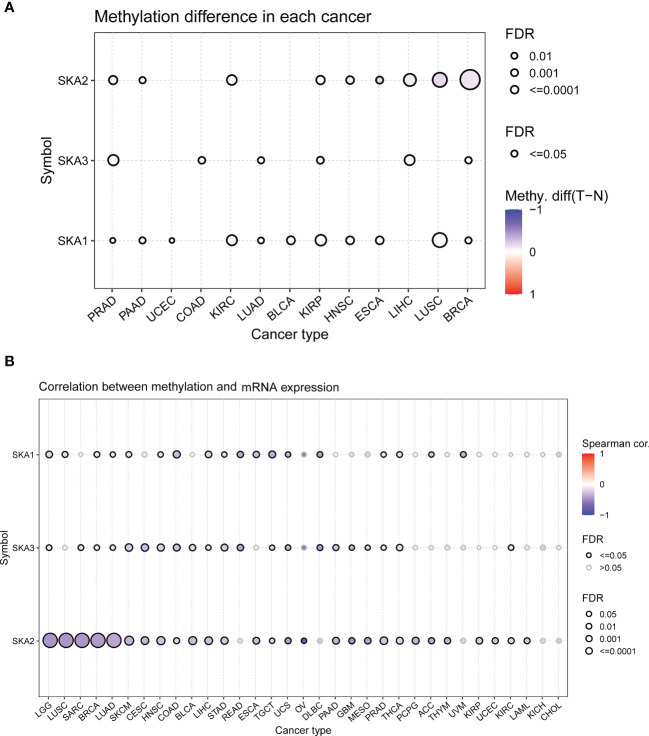
The methylation information of SKA1/2/3 in the indicated tumor types. **(A)** DNA methylation patterns in tumor and normal samples of 13 cancer types. The bubble size represents the false discovery rate (FDR), and the bubble color represents the fold-change. The increased or decreased methylation are presented with red dots or blue dots, respectively. A deeper color indicates a larger difference. **(B)** The relationship of methylation with mRNA levels of SKA1/2/3. The darker is the color, the stronger is the correlation.

To explore the mutation status of SKA family genes in various cancer types, we investigated the SNV profile using GSCALite (24). SKA3 was identified as a deleterious mutated gene with the highest frequency in UCEC according to the heatmap of SNV percentage (**Figure 3A**). We next assessed the copy number variants (CNVs) of SKA1/2/3 across cancers. The distribution proportion for the different CNV types, including heterozygous amplification, heterozygous deletion, homozygous amplification, and homozygous deletion, was computed with each SKA family gene across 33 cancer types (**Figure 3B**). The landscape of CNVs of the three SKA family genes is heterozygous amplification and heterozygous deletion. **Figure 3C** shows a positive relationship between the CNV of SKA2 and its mRNA value in 22 of 33 cancer types. The mRNA level of SKA family genes was positively related to the CNV of homozygous or heterozygous amplification but negatively related to the CNV of homozygous or heterozygous deletion (**Figures 3D, E**). Furthermore, **Figure 4A** shows the methylation landscape of SKA1/2/3 in 33 cancer types. We found that the methylation levels of SKA1/2/3 differed among 13 cancer types (**Figure 4A**). Studies have generally confirmed that the DNA methylation level of genes is negatively correlated with the mRNA expression of genes (36, 37). A general negative correlation was observed between the methylation levels of SKA1/2/3 and their mRNA levels (**Figure 4B**). Our results also confirmed this phenomenon.

### Survival analysis according to the SKA score

**Figure 5:**
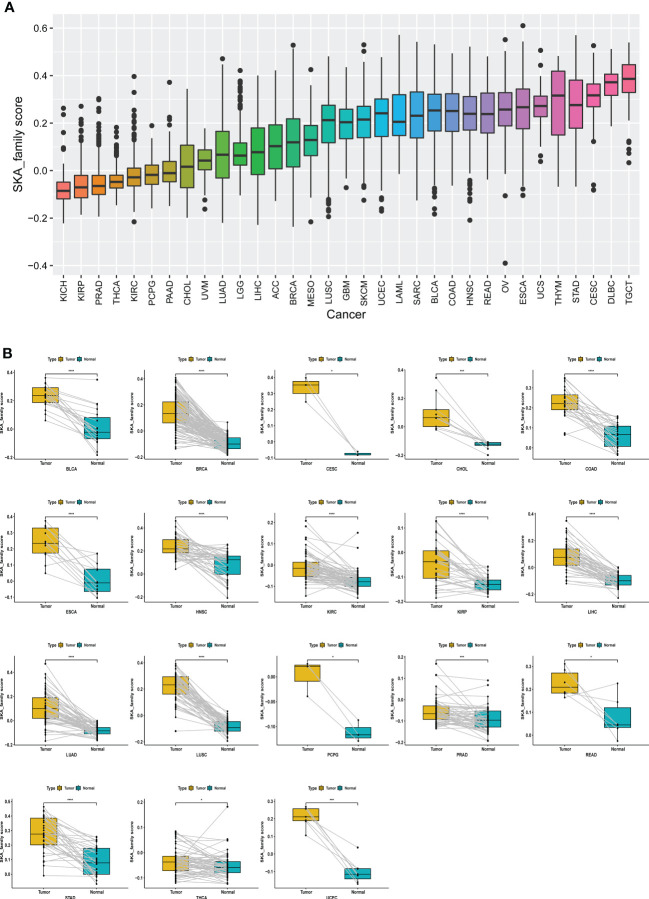
Establishing a scoring system across cancers. **(A)** Rank-order plot of SKA scores from lowest to highest across cancers. **(B)** The difference analyses of SKA score in tumor tissue samples vs. paired adjacent normal tissue samples in indicated cancer types. *P < 0.05, ***P < 0.001, ****P < 0.0001.

Considering the individual heterogeneity and complexity of SKA family genes, based on ssGSEA, we constructed a scoring system to quantify the SKA gene modification pattern of individual patients in each cancer type in the TCGA cohort, termed the SKA score. As shown in **Figure 5A**, TGCT had the highest SKA score, while KICH had the lowest SKA score. In addition, the SKA score was found to be elevated in cancer tissues, including BLCA, BRCA, CESC, CHOL, COAD, ESCA, HNSC, KIRC, KIRP, LIHC, LUAD, LUSC, PCPG, PRAD, READ, STAD, THCA, and UCEC, compared with the cancer adjacent tissues (**Figure 5B**).

**Figure 6:**
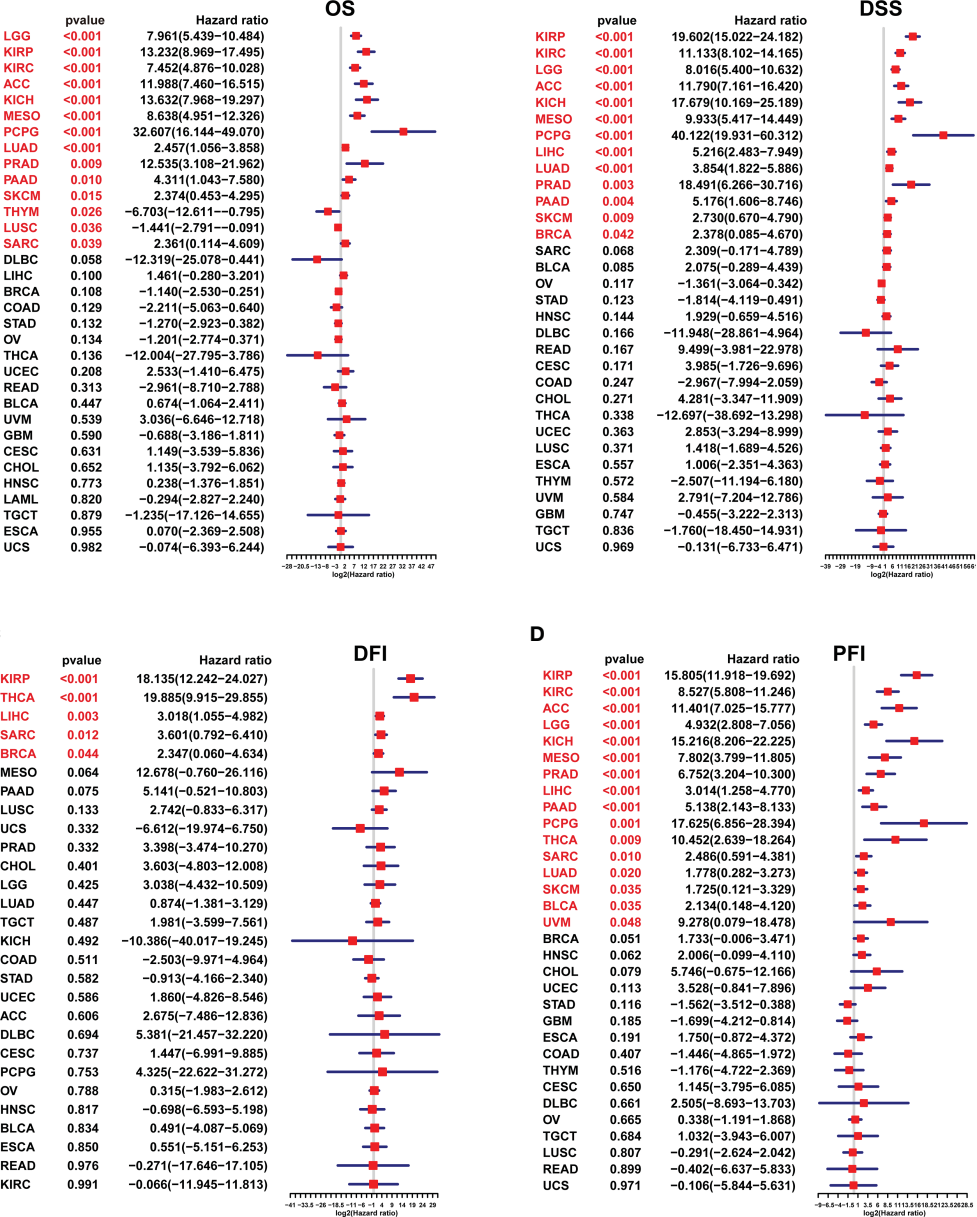
The survival analysis of the SKA score. **(A–D)** Forest plots of the Cox analysis results of the SKA score across cancers. **(A)** OS, **(B)** disease-specific survival, **(C)** disease-free interval, and **(D)** progression-free interval.

With univariate Cox regression, we investigated the prognostic value of the SKA score by analyzing the association of the SKA score with the four survival outcomes OS, DSS, DFI and PFI. For the univariate results in pan-cancer: (1) For OS, SKA score was a significant risk factor in KIRP (HR=13.232), LGG (HR=7.961), KIRC (HR=7.452), ACC (HR=11.988), KICH (HR=13.632), MESO (HR=8.638), PCPG (HR=32.607), LUAD (HR=2.457), PRAD (HR=12.535), PAAD (HR=4.311), SKCM (HR=2.374) and SARC (HR=2.361), whereas a protective factor in THYM (HR=-6.703) and LUSC (HR=-1.441) (**Figure 6A**); (2) For DSS, SKA score was a significant risk factor in KIRP (HR=19.602), KIRC (HR=11.133), LGG (HR=8.016), ACC (HR=11.790), KICH (HR=17.679), MESO (HR=9.933), PCPG (HR=40.122), LIHC (HR=5.216), LUAD (HR=3.854), PRAD (HR=18.491), PAAD (HR=5.176), SKCM (HR=2.730) and BRCA (HR=-2.378) (**Figure 6B**); (3) For DFI, SKA score was a risk factor in KIRP (HR=18.135), THCA (HR=19.885), LIHC (HR=3.018), SARC (HR=3.601), BRCA (HR=2.347) (**Figure 6C**). The sample size with DFI information in **Figure 6C** was smaller and therefore had fewer positive results; (4) For PFI, SKA score was a significant risk factor in KIRP (HR=15.805), KIRC (HR=8.527), ACC (HR=11.401), LGG (HR=4.932), KICH (HR=15.216), MESO (HR=7.802), PRAD (HR=6.752), LIHC (HR=3.014), PAAD (HR=5.138), PCPG (HR=17.625), THCA (HR=10.452), SARC (HR=2.486), LUAD (HR=1.778), SKCM (HR=1.725), BLCA (HR=2.134), and UVM (HR=9.278) (**Figure 6D**). From these figures, we know that in terms of prognosis, the SKA score can be used as a potential prognostic risk factor in 11 cancer types, including KIRP, LGG, KIRC, ACC, KICH, MESO, PCPG, LUAD, PRAD, PAAD, and SKCM. Taken together, these findings revealed that the SKA score was a robust risk factor for numerous cancer types and displayed a strong HR value at the pan-cancer level.

### GSVA of SKA score

**Figure 7:**
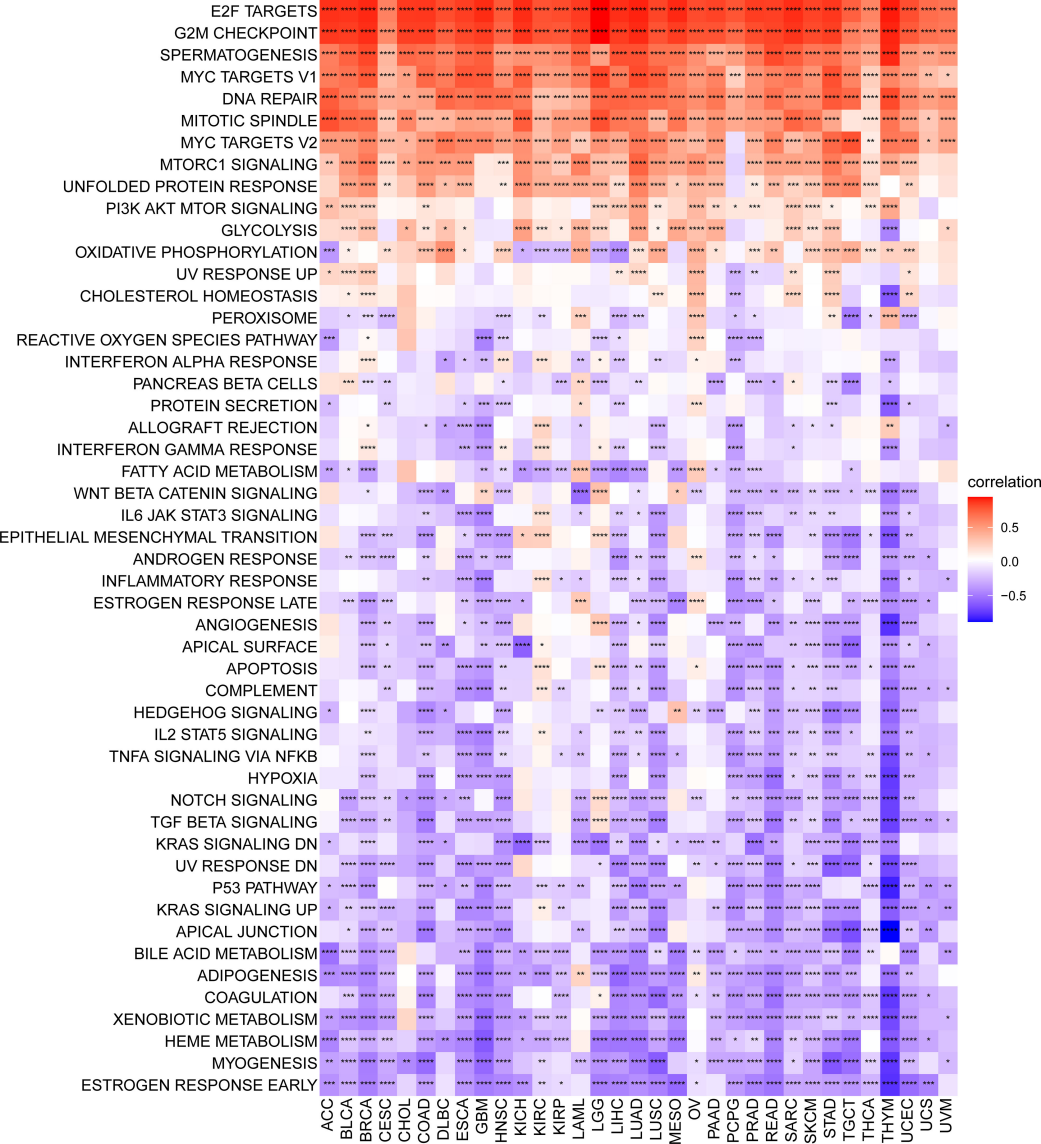
Gene set variant analysis of the SKA score. Heatmap of the relationship of the SKA score with the 50 HALLMARK pathway score across 33 cancer types. *P < 0.05, **P < 0.01, ***P < 0.001, ****P < 0.0001.

We conducted a GSVA to investigate the potential pathways likely affected by the SKA score according to 50 HALLMARK pathways. **Figure 7** shows the correlation of the SKA score with the GSVA pathways. The obtained results showed that the SKA score was positively correlated with cell proliferation-related pathways and DNA replication in pan-cancer, such as E2F targets, G2M checkpoint, MYC targets V1/V2, mitotic spindle and DNA repair, which suggested that tumor cells might have a strong proliferation ability through those signaling pathways. To better explore the pathways SKA family genes may participate in, we also conducted GSEA using R package “clusterprofiler.” We observed that SKA family genes were mainly enriched in cell cycle related pathways in most tumor types, including ACC, GBM, LGG, LIHC, LUSC, and UVM (**Figures S1A–P**). In the tumor microenvironment, these pathways are involved in tumorigenesis by mediating proliferation, migration, invasion, and metastasis. Additionally, the obtained results indicated that the SKA score was negatively related to immune-related pathways, such as the TGF beta signaling pathway, IL6-JAK-STAT3 signaling pathway and p53 pathway. Therefore, we speculate that patients in the high SKA score group may exert weaker tumor immunity, which may be one of the reasons for the poor prognosis of this group of patients.

### Relationship between SKA score and the TME

**Figure 8:**
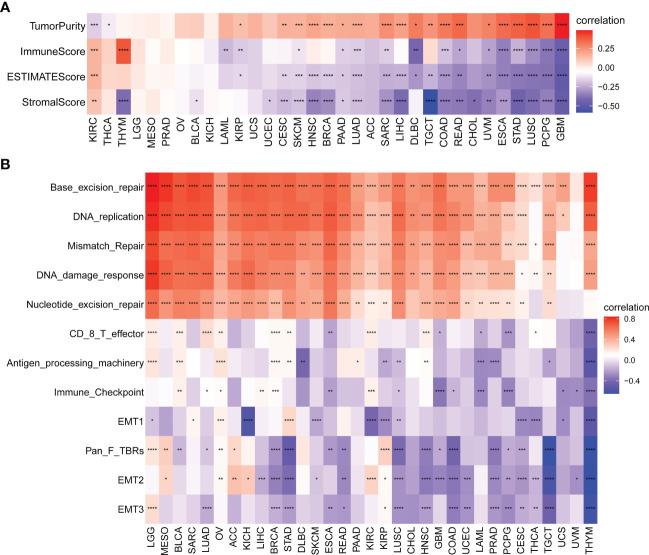
Tumor microenvironment (TME) analysis of the SKA score. **(A, B)** Heatmap depicting the relationship of the SKA score with **(A)** TME scores (including immune score, stromal score, ESTIMATE score, and tumor purity) and **(B)** TME-related pathways (including immune-related pathways, stromal-related pathways, and DNA repair-related pathways) across 33 cancer types. *P < 0. 05, **P < 0.01, ***P < 0. 001, ****P < 0.0001.

We further explored the relationship between the SKA score and TME score (including stromal, immune and ESTIMATE scores) in 33 cancer types. We found that in most cancer types, the SKA score was negatively related to the stromal score, immune score, and ESTIMATE score but was positively associated with tumor purity (**Figure 8A**). In the TME, the SKA score may suppress antitumor immunity and promote tumor cell proliferation, survival, and invasion. Next, we extracted and computed TME-related pathway scores from a published paper (28). Then, we explored the relationship between the SKA score and TME-related pathways. We also found that the SKA score was closely positively correlated with DNA replication, mismatch repair and DNA damage response but negatively correlated with immune and stromal-related pathways, including EMT1/2/3, CD8T effector, and immune checkpoint, in 33 cancer types (**Figure 8B**). Both analyses were similar.

### Immune infiltrating analysis

**Figure 9:**
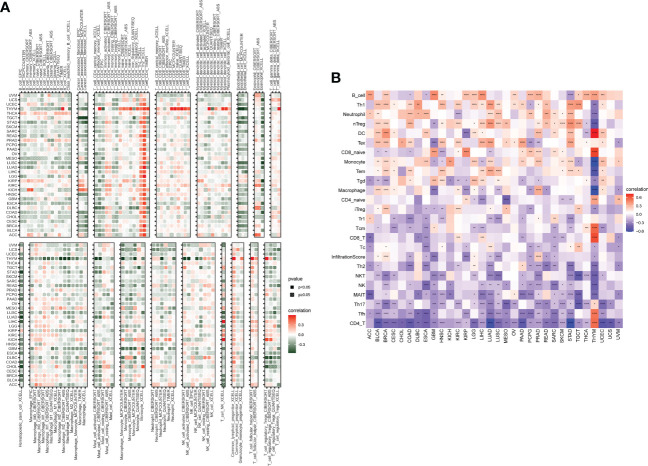
Immune cell infiltrate analysis. **(A, B)** A strong relationship was observed between multiple immune cells and the SKA score based on the **(A)** TIMER2 database or **(B)** ImmuCellAI. *P < 0.05, **P < 0.01, ***P < 0.001, ****P < 0.0001.

The abovementioned results revealed that the SKA score was closely associated with the immune score. Thus, we further investigated the relationship between the SKA score and immune cells in the TME. The obtained results revealed that the SKA score was negatively associated with most immune cells across cancers according to the TIMER2 database, indicating an immune-suppressive TME (**Figure 9A**). The results from the ImmuCellAI database revealed that the SKA score was also generally connected to immune cell infiltration in the TME at the pan-cancer level (**Figure 9B**). The results of the two analyses are consistent and mutually verifiable.

### The correlation of SKA score with immunotherapy response

**Figure 10:**
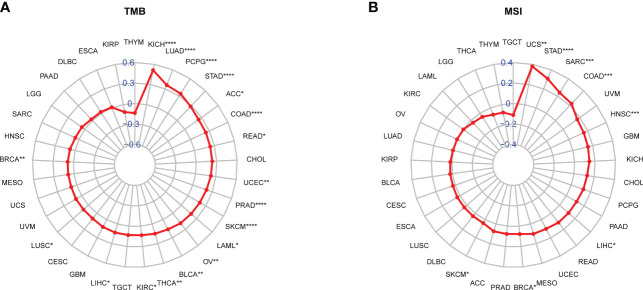
Correlation between the SKA score and TMB and MSI values. **(A, B)** Radar plot of the correlation between the SKA score and **(A)** TMB or **(B)** MSI. *P < 0.05, **P < 0.01, ***P < 0.001, ****P < 0.0001.

Immunotherapies targeting PD-1/PD-L1 have made great clinical progress in immune checkpoint therapy (38–40). Elevated tumor mutation burden (TMB) or microsatellite instability (MSI) values have emerged as strong markers in predicting the immune checkpoint inhibitor (ICI) response (41–43). The correlation between the SKA score and immunotherapy-related biomarkers (TMB and MSI) was further explored. The obtained results revealed that the SKA score was positively associated with TMB in 18 cancer types as well as MSI in 8 cancer types (**Figures 10A, B**). A significant correlation was observed between the SKA score and the TMB value of SKCM and BLCA, and we speculate that patients with high SKA scores are sensitive to immunotherapy in some cancer types.

**Figure 11:**
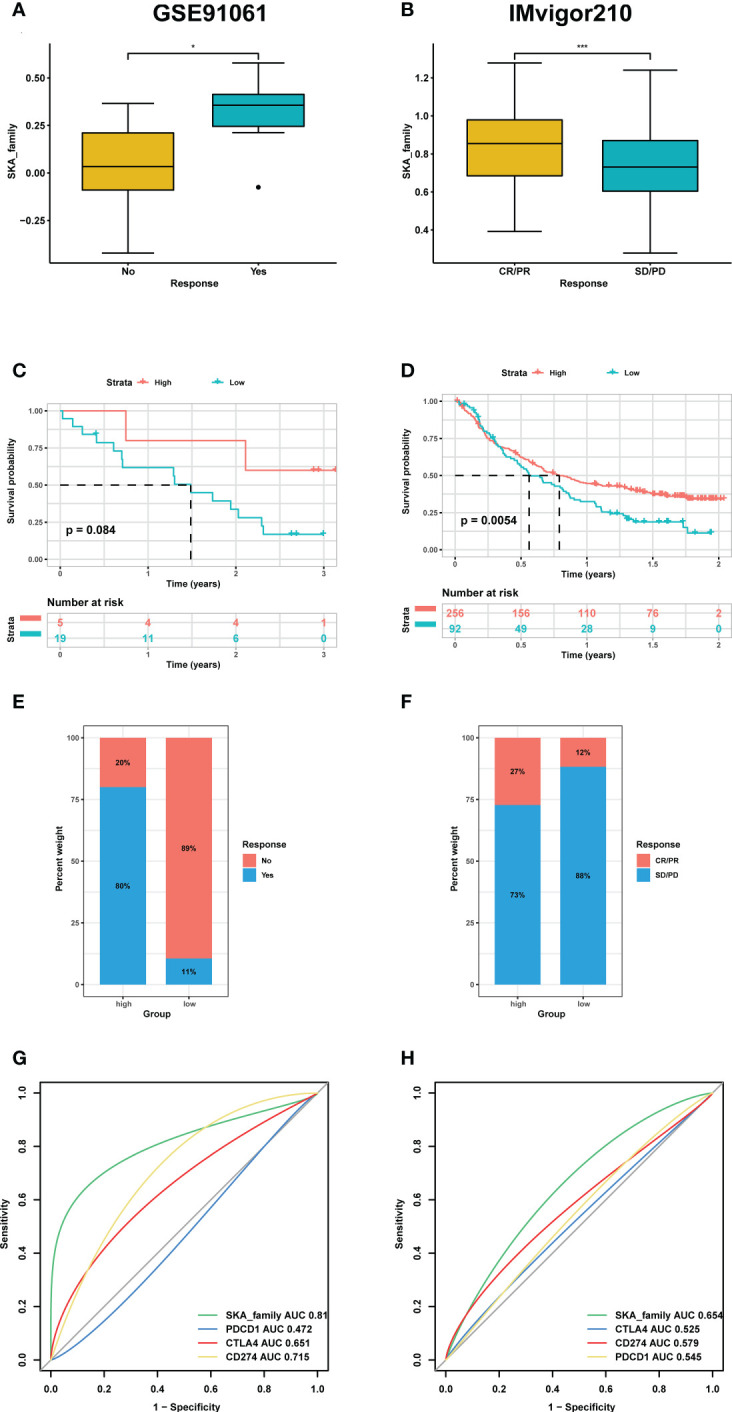
The association between SKA score and immunotherapy response. **(A, E)** Differences in the m6Ascore among distinct anti-PD-1/L1 clinical response groups in the GSE91061 **(A)** and IMvigor210 cohorts **(E)**. **(B, F)** Kaplan–Meier survival analysis of the SKA score in the GSE91061 **(B)** and IMvigor210 cohorts **(F)**. **(C, G)** The percentage of responsive and progressive patients in the high- and low-SKA score groups in the GSE91061 **(C)** and IMvigor210 cohorts **(G)**. **(D, H)** The predictive ability of the SKA score in patients receiving anti-PD-1/L1 therapy in the GSE91061 **(D)** and IMvigor210 cohorts **(H)**. CR, complete response; PD, progressive disease; PR, partial response; SD, stable disease. *P<0.05, ***P<0.001.

To validate our hypothesis, we screened independent immunotherapy-related datasets from the SKCM (GSE91061 cohort) and BLCA (IMvigor210 cohort) datasets and computed the corresponding SKA score for each patient. We investigated whether the SKA score could predict patients’ response to immune checkpoint blockade therapy based on two immunotherapy cohorts. In this study, a total of 24 melanoma samples were included in the GSE91061 cohort, and 348 BLCA samples were included in the IMvigor210 cohort. In both the anti-PD-1 cohort (GSE91061) and the anti-PD-L1 cohort (IMvigor210), patients with high SKA scores exhibited a significantly prolonged clinical response (**Figures 11A, B**) and markedly prolonged survival (**Figures 11C, D**). The significant therapeutic advantages and clinical response to anti-PD-1/L1 immunotherapy in patients with high SKA scores compared to those with low SKA scores were confirmed. Meanwhile, higher objective response rates were observed in the patients with high SKA scores than in those with low scores (**Figures 11E, F**). In addition, the predictive value of the SKA score in patients treated with anti-PD-1/L1 immunotherapy was superior to those of CTLA4 and PD-1/L1 (**Figures 11G, H**). This was another confirmation that patients with relatively high SKA scores were more suitable for immunotherapy. The abovementioned results of a series of observations and validations implied that the SKA score may serve as a potential predictor of immunotherapy efficacy and chemotherapy efficacy for tumor therapy.

### Immunohistochemistry

**Figure 12:**
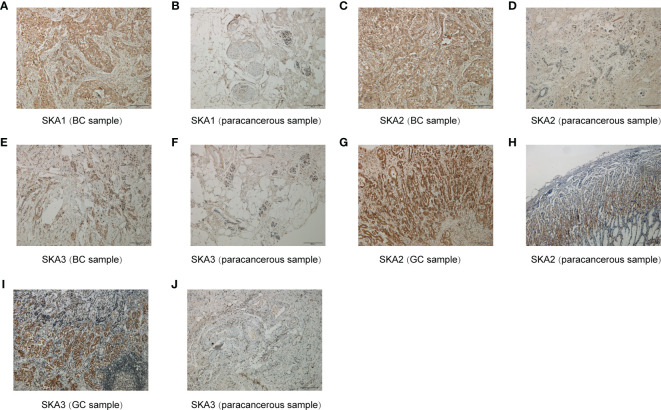
Immunohistochemical results for SKA1 **(A, B)**, SKA2 **(C, D, G, H)** and SKA3 **(E, F, I, J)**. Hematoxylin stained the cell nucleus blue, and positive expression of DAB is brownish yellow. DAB, diaminobenzidine; BC, breast cancer; GC, gastric cancer; Scale bar=200mm.

To further validate the results for SKA1/2/3 expression, immunohistochemical staining was performed. The results confirmed that SKA1/2/3 were higher in BC tissues than that in adjacent normal tissues, and SKA2 and SKA3 were higher in GC tissues than that in adjacent normal tissues (**Figures 12A–J**).

### Identification of potential small molecule drugs

We first selected the top 30 small molecule drugs targeting SKA family genes (|r|> 0.3). The obtained results revealed that the expression of SKA1/2/3 was positively associated with six cancer drugs but negatively correlated with the IC50 of 24 cancer drugs in GDSC (**Figure S2A**). This finding suggested that SKA family genes may be associated with tumor drug resistance in these six cancer drugs, such as trametinib and selumetinib. Our CTRP results also showed that SKA3/1 was negatively correlated with the IC50 of 30 cancer drugs (**Figure S2B**). The lower was the IC50, the higher was the sensitivity to the drugs. These findings suggested that SKA family genes may be correlated with cancer sensitivity to multiple drugs.

## Discussion

Cancer is a multifactorial disease that is influenced by multiple factors such as intracellular metabolism, evasion of immune surveillance, dysregulation of the cell cycle and inflammation (44). SKA1/2/3 contributes to the stability of mitotic metaphase (45). Several studies have suggested that SKA1/2/3 are involved not only in mitosis but also in apoptosis and tumor development. The dysregulation of SKA1/2/3 expression is a common phenomenon in malignant tumors, indicating that the SKA family is significantly related to malignancy. Currently, the function and mechanisms of SKA1/2/3 in the development and progression of certain cancers are being increasingly studied. Nevertheless, an understanding of the biological regulation of SKA family genes across cancers is lacking.

In this study, based on the ssGSEA method, we constructed the SKA score to quantify the SKA gene modification pattern of individual patients. We investigated the differential expression and potential prognostic value of the SKA score at the pan-cancer level. In multiple cancer types, including BLCA, BRCA, CESC, CHOL, COAD, ESCA, HNSC, KIRC, KIRP, LIHC, LUAD, LUSC, PCPG, PRAD, READ, STAD, THCA, and UCEC, the SKA score was significantly elevated in tumor tissues compared with normal tissues. Results of Immunohistochemistry shown that SKA1/2/3 proteins were highly expressed in BC or GC tissues. Subsequent analysis showed that the SKA score was closely associated with tumor development and served as a strong risk factor for four survival outcomes (OS, DSS, PFI, and DFI) across 33 cancer types. The precise molecular mechanisms of SKA family genes in the development and progression of cancer are not completely clear and require additional studies. Nevertheless, the association between SKA family genes and some underlying tumor-related pathways may provide several valuable clues to understand the unique functional mechanisms in cancers. An in-depth analysis of SKA family genes confirmed that the SKA score was positively related to some cell cycle pathways and DNA replication in pan-cancer, including E2F targets, G2M checkpoint, MYC targets V1/V2, mitotic spindle and DNA repair. The cell cycle is an evolutionarily conserved, highly coordinated process that is essential to cell growth. Aberrations of the cell cycle are a hallmark of cancer (46). Remarkably, cyclin-dependent kinase 1 (CDK1) is an important cell cycle regulator that is involved in the progression of the cell cycle (47). Interestingly, a previous study found that mutant SKA3 lacking CDK1 phosphorylation failed to locate KT (48). These findings, combined with our observations, hint at the potential therapeutic target of the SKA family in cancer treatment.

The TME plays a role in promoting tumor development, metastasis, and resistance to chemotherapy and immunotherapy (49, 50). Given the importance of the TME in cancer, we next investigated the association between the SKA score and the TME, and the obtained results revealed that both the immune score and stromal score were negatively related to the SKA score, which indicated that cancer tissues with high SKA scores had significantly higher tumor purity and lower immune scores than those with low SKA scores. To validate these findings, we further analyzed the correlation of TME-related pathways with the SKA score based on a published paper, and the obtained results also showed that the SKA score was positively correlated with cell cycle pathways, DNA replication and repair pathways but negatively correlated with immune- or stromal-related pathways, which was consistent with the abovementioned findings. We next explored the association of the SKA score with immune cell infiltrates and observed that in 33 cancer types, the SKA score was closely associated with multiple immune cells, representing a state of immune-suppressed TME. The abovementioned results showed that patients with high SKA scores had poor immune cell infiltration, which may have contributed to the worse prognosis in cancer patients. Recent evidence from many studies has shown that immunotherapy, such as anti-PD-1/L1 therapy, has emerged as the most eye-catching treatment method for malignant tumors, and the low response rate in the clinic is a great obstacle to the development of ICI therapy. In addition to PD-L1, high TMB and high MSI have been shown to be useful biomarkers for better immunotherapy response in cancer (41, 42, 51). High TMB levels lead to an increase in tumor neoantigens, which may trigger the immune system to attack the tumor. The reasons why high MSI and high TMB predict the response to immunotherapy are probably related because high MSI almost inevitably leads to a high TMB. To explore the correlation of the SKA score with ICI response, we conducted Spearman correlation analysis, and the obtained results showed that the TMB of 18 types of cancers as well as the MSI of 7 types of cancers, including SKCM and BLCA, were all positively correlated with the SKA score. Therefore, combining the abovementioned information, we speculate that patients with high SKA scores are sensitive to anti-PD-1/L1 treatment response. This hypothesis was validated in independent cohorts of BLCA (IMvigor210) and SKCM (GSE91061) patients who received anti-PD-L1/PD-1 therapy. Thus, our results confirm that a high SKA score is closely related to the TME in a variety of cancers and that an elevated SKA score may serve as a novel tool in patients receiving anti-PD-1/L1 therapy, especially in patients with BLCA and SKCM. At the same time, our study also demonstrated a close association of SKA1/2/3 and drug sensitivity across cancers, suggesting a potential of SKA family genes and related pathways of biological functions as therapeutic targets.

Compared with previous studies on certain cancers, our study has three strengths. First, previous studies aimed to develop and validate a prognostic signature in certain cancers. Our study focused on pan-cancer data, making the SKA scoring system more universal and applicative. Second, compared with the studies assessing only one survival outcome (usually OS), the SKA score in our study did not merely focus on four survival outcomes (OS, DSS, PFI, and DFI) across 33 cancer types but displayed a strong HR value for cancers. Third, we believe that this study was the first to explore the correlation of the SKA score with immunotherapy response based on the SKCM and BLCA cohort data, which had not been reported in certain cancer types.

## Conclusion

Collectively, our results reveal that the SKA score is a robust risk factor for multiple cancer types and plays central roles in tumor development. A high SKA score was closely correlated with a variety of immune cells of the TME across multiple cancer types. Moreover, the SKA score can be used as a potential predictive biomarker for patients receiving anti-PD-1/L1 therapy. Our comprehensive analysis highlighted the potential clinical value of SKA1/2/3-related strategies for cancer treatment, which highlights their significance for clinical practice and guidelines.

## Data availability statement

The original contributions presented in the study are included in the article/**Supplementary Material**. Further inquiries can be directed to the corresponding authors.

## Ethics statement

The studies involving human participants were reviewed and approved by the ethics committee of the First Affiliated Hospital of Guangxi Medical University (approval No.2022-E434-01). Written informed consent for participation was not required for this study in accordance with the national legislation and the institutional requirements.

## Author contributions

GD contributed to conception and design of the study. HW contributed to the experimental design and performed the experiments. ZL and LH analyzed the data and wrote the first draft of the manuscript. JL, WY, WL, QL, XD, and HW wrote sections of the manuscript. All authors contributed to the article and approved the submitted version.

## Supplementary material

Supplementary Figure 1 GSEA of SKA1/2/3 in TCGA pan-cancer. **(A–P)** The top 20 GSEA results of SKA1/2/3 in indicated tumor types from TCGA.

Supplementary Figure 2 Associations between SKA1/2/3 and drug sensitivity.Associations between SKA1/2/3 and drug sensitivity. **(A, B)** The relationship of drug sensitivity of GDSC (top 30) and CTRP (top 30) with SKA1/2/3 mRNA expression. Pearson correlation analysis was performed to obtain the correlation between gene mRNA expression and drug IC50. The P-value was adjusted by FDR. Blue bubbles represent negative correlations, red bubbles represent positive correlations, and the deeper is the color, the higher is the correlation.

Supplementary Table 2 Abbreviations and sample size for 33 cancer types.
