# Supplementary figures and images for: Immunological role and prognostic value of the SKA family in pan-cancer analysis
Source: Front Immunol. 2023 Apr 26;14:1012999. doi: 10.3389/fimmu.2023.1012999 (PMC10169755; doi:10.3389/fimmu.2023.1012999)

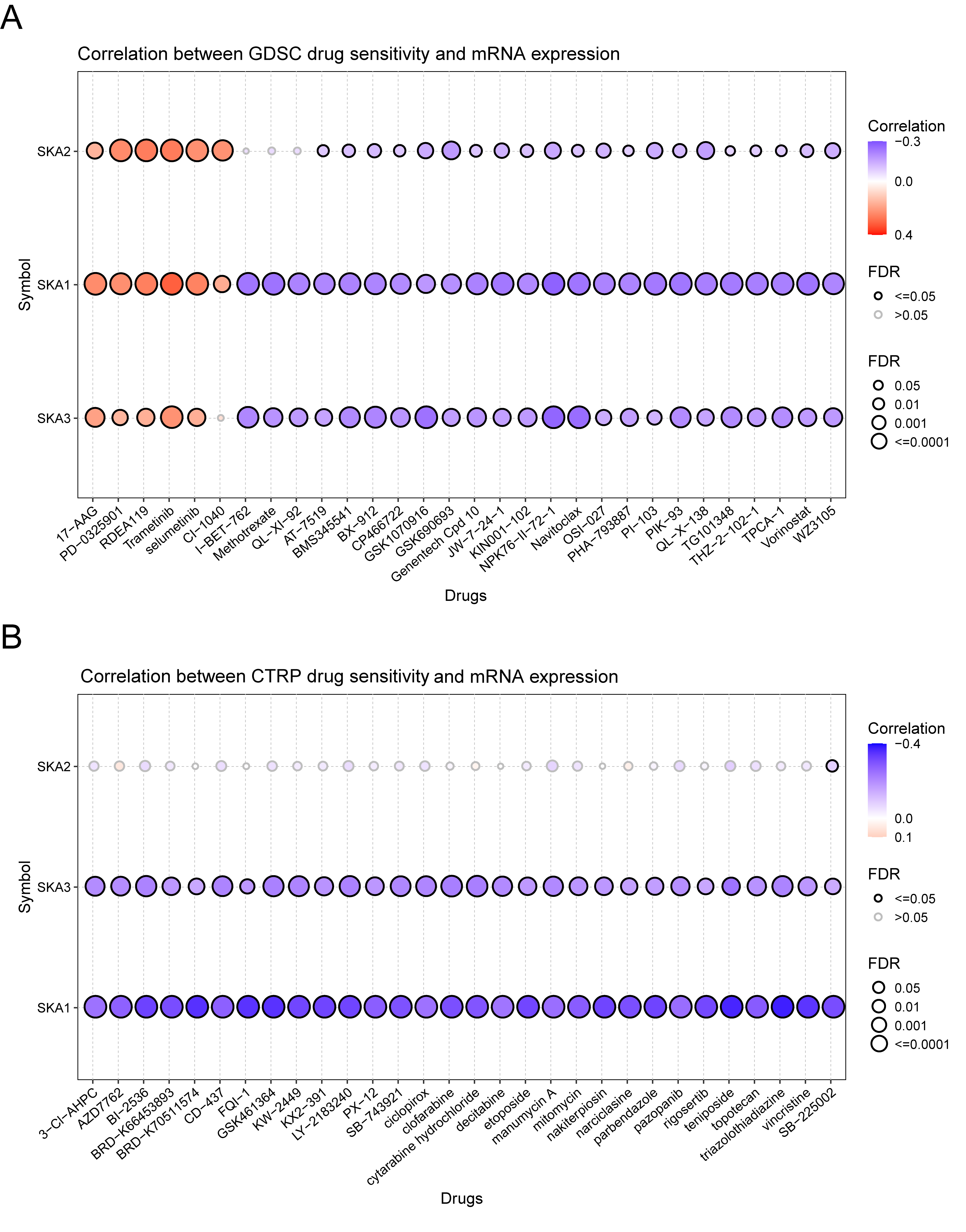

Supplement: Supplementary Figure 2 — Associations between SKA1/2/3 and drug sensitivity.Associations between SKA1/2/3 and drug sensitivity. (A, B) The relationship of drug sensitivity of GDSC (top 30) and CTRP (top 30) with SKA1/2/3 mRNA expression. Pearson correlation analysis was performed to obtain the correlation between gene mRNA expression and drug IC50. The P-value was adjusted by FDR. Blue bubbles represent negative correlations, red bubbles represent positive correlations, and the deeper is the color, the higher is the correlation. [file Image_2.tif]
