# Supplementary material for: Immunological role and prognostic value of the SKA family in pan-cancer analysis
Source: Front Immunol. 2023 Apr 26;14:1012999. doi: 10.3389/fimmu.2023.1012999 (PMC10169755; doi:10.3389/fimmu.2023.1012999)
Supplement: Supplementary file 3 [file Table_1.docx]

**Table S1. The R script of SKA score in this study.**

**##########**

library(GSVA)

library(limma)

library(GSEABase)

setwd("C:\\Users\\lizhengtian\\Desktop\\SKA score")

files=dir()

files=grep("^symbol.", files, value=T)

outTab=data.frame()

for(i in files){

CancerType=gsub("symbol\\.|\\.txt", "", i)

rt=read.table(i, header=T, sep="\t", check.names=F)

rt=as.matrix(rt)

rownames(rt)=rt[,1]

exp=rt[,2:ncol(rt)]

dimnames=list(rownames(exp),colnames(exp))

mat=matrix(as.numeric(as.matrix(exp)),nrow=nrow(exp),dimnames=dimnames)

mat=avereps(mat)

mat=mat[rowMeans(mat)>0,]

geneSet=getGmt("SKA.gmt", geneIdType=SymbolIdentifier())

#ssgsea analysis

ssgseaScore=gsva(mat, geneSet, method='ssgsea', kcdf='Gaussian', abs.ranking=TRUE)

group=sapply(strsplit(colnames(mat),"\\-"), "[", 4)

group=sapply(strsplit(group,""), "[", 1)

Type=ifelse(group==0, "Tumor", "Normal")

outTab=rbind(outTab, cbind(t(ssgseaScore), Type, CancerType))

}

out=cbind(ID=row.names(outTab), outTab)

write.table(out, file="SKA score.txt", sep="\t", quote=F, row.names=F)
